# Assessing Oral Health-Related Quality of Life in Older Tennessean Adults

**DOI:** 10.3390/dj13050203

**Published:** 2025-05-01

**Authors:** Yeleeya Y. Li, Ying Liu, Memunat Ogunmefun, Kesheng Wang

**Affiliations:** 1 Department of Biology, College of Liberal Arts and Sciences, University of Florida, Gainesville, FL 32611, USA; yeleeyal@gmail.com; 2 Department of Biostatistics, College of Public Health, East Tennessee State University, Johnson City, TN 37614, USA; ogunmefunm@etsu.edu; 3 Department of Biobehavioral Health & Nursing Science, College of Nursing, University of South Carolina, Columbia, SC 29208, USA; kesheng@mailbox.sc.edu

**Keywords:** quality-of-life, oral health, factor analysis, gerodontology, geriatrics, psychosocial well-being

## Abstract

**Background**: Tennessee has one of the worst rankings for older adults’ oral health in the United States. This study aims to evaluate the oral health-related quality of life (OHRQoL) among older individuals (aged 60 and above) in Tennessee using the Oral Health Impact Profile-14 (OHIP-14) questionnaire. **Methods**: The data were collected from the 233 Tennessee Smile-on program participants in the early phase of the COVID-19 pandemic, between December 2019 and August 2021. The frequency and percentage for each subgroup were calculated. Cronbach’s alpha was used to measure the internal consistency or reliability of OHIP in this study. Factor Analysis (FA) with oblique rotation was conducted to explore the underlying factor structure of the OHIP questionnaire set. A *p* < 0.05 was considered statistically significant. **Results**: The majority of participants were retired (59.66%), and there was a significant difference in OHIP_sum scores among different employment statuses (*p* = 0.018). Cronbach’s alpha showed the domains of psychological discomfort, physical disability, and psychological disability were highly correlated with the total score (alpha = 0.8). Factor analysis identified three main dimensions: physical discomfort, psychological distress, and functional disability, and they can explain over 90% of the total variance. Individuals measure of sampling adequacy (MSA) and overall MSA are greater than 0.9, indicating excellent sampling adequacy. **Conclusions**: The study suggested that oral health can be assessed not only through examinations by dental professionals but also by considering emotional and social well-being. However, a limitation of the study is that it was conducted during the COVID-19 outbreak, which restricted participant involvement.

## 1. Introduction

With the development of technology and medical science, people can live significantly longer than before, which leads to an increasing population of older adults. The World Health Organization (WHO) predicted that the percentage of senior individuals (older than 60) will increase from 12% in 2015 to 22% in 2050 [1]. It is reported that older adults accounted for 17.3 of the American population, with a total of 57.8 million people aged 65 years or older, including 31.9 million women and 25.9 million men in 2022 [2]. The older population has been increasing quickly in the United States. It can be expected that older adults (aged 65 years and above) will reach roughly 78.3 million Americans by 2040 [3]. Tennessee Department of Health reported there were 1.6 million people aged 60 and older in 2024, comprising roughly 25% of Tennessee’s population [4]. The state’s population of older adults is projected to grow roughly 30%, from 1.6 million in 2020 to 2.1 million in 2040 [5].

Aging in any population can be viewed as an achievement as it signifies the result of successful prevention and control of diseases as well as socio-economic development. It is also perceived as a challenge as the increasing number of older people in society necessitates adjustments in social and healthcare systems [6]. Due to an increase in life expectancy over the years, there has been a shift in the prevalence of infectious diseases, with chronic health problems mostly associated with aging [7]. Older individuals often suffer from chronic diseases that affect the cardiovascular, respiratory, and central nervous systems, along with metabolic disorders and cancer [8].

The susceptibility of older adults to chronic and degenerative diseases makes them more vulnerable to oral diseases as conditions like diabetes increase periodontitis incidence by 86%, rheumatoid arthritis complicates oral care, and multiple medications for non-communicable diseases cause xerostomia, leading to further oral problems such as dental caries, periodontal disease, and infections, which can result in tooth loss [9]. The incidence of chronic diseases and frequent long-term use of multiple medications often lead to hyposalivation, increasing the risk for caries and mucosal infections. This can contribute to tooth loss, which further causes age-related vertical dimension loss and poor masticatory function [10]. This contributes to poor eating habits in older adults, as loose, painful teeth or ill-fitting dentures as a result of vertical dimension loss can reduce their desire or ability to eat and compromise their nutritional status, which can further undermine oral health [11].

Furthermore, many studies have revealed a significant association between dental health and age-related frailty, suggesting that oral health might be a predictor of age-related frailty [12]. Despite the well-established connection between chronic diseases, such as cardiovascular and neoplastic disorders, and oral health, there has been significant neglect of oral health issues among older people [10].

The oral health-related quality of life (OHRQoL) is a multifaceted concept that captures people’s perceptions about important factors in their daily lives [13]. It encompasses various interrelated domains that are considered vital in clinical decision-making and further complement clinical outcomes. Assessment of OHRQoL shifts the focus of dental care to individual social and environmental factors that can influence oral health [14]. Objective evaluations of oral diseases often fail to correctly reflect patients’ perceptions of their oral health. OHRQoL assessment tools are valuable for evaluating oral health from an individual’s perspective by considering functional and psychosocial impacts and assessing changes in oral status during care while integrating patient perceptions and expectations [15]. In addition to understanding individual perceptions and feelings, the evaluation of quality of life (QoL) supports public health policy planning by identifying population needs and care objectives and aids in research by assessing treatment outcomes and developing evidence-based strategies [16]. In clinical practice, information on OHRQoL aids in developing patient-centered treatment plans that consider individuals’ unique life contexts and personal perceptions of health, emphasizing both health promotion and disease elimination [17].

There are several tools used to measure OHRQoL, such as the Geriatric Oral Health Assessment Index (GOHAI), Oral Impacts on Daily Performances (OIDP), and the Oral Health Impact Profile (OHIP-14) commonly used by researchers and clinicians [17,18]. The OHIP-14 is a shorter version of the OHIP-49 that assesses seven dimensions of the impacts of oral diseases on people’s OHRQoL including physical pain, functional limitation psychological discomfort, psychological disability, physical disability, social disability, and handicap [19].

In modern society, while people are living longer and the older population is growing rapidly, changes in social structures have created many challenges for older adults. These include financial difficulties, estrangement from their children, the loss of a partner, family members, and friends, feelings of purposelessness and indignity, functional decline, adaptation problems, high healthcare costs, ageism, loneliness, and digital exclusion [20,21]. In addition to these, older individuals often experience multimorbidity and polypharmacy, which complicate treatment plans and increase vulnerability to adverse outcomes. Cognitive decline—including mild cognitive impairment and dementia—affects decision-making, independence, and quality of life. Mobility limitations, poor oral health, and difficulties in accessing transportation and community services further hinder daily functioning and social engagement [20,21]. Many older individuals also face stigmatization related to disability or dependency, leading to social withdrawal and reduced self-worth [21].

Assessing the subjective perspective of older people about their oral and general health requires the use of validated tools to examine oral health’s impact on physical and psychosocial well-being, measure oral treatment needs, and evaluate the impact of dental treatment [22].

In terms of oral health, Tennessee consistently has one of the lowest ranks of US states due to several interrelated factors. Traditional health insurance, such as Medicare and Tennessee’s state Medicaid program, does not cover dental benefits [23]. Tennessee is also among the least educated states in the nation, with only 30.5% of adults aged 25 and older holding a bachelor’s degree or higher, compared to the national average of 35%. Additional barriers to dental care include limited public transportation and high poverty rates [24]. However, studies focusing on the senior population in Tennessee are lacking. This study aims to evaluate the OHRQoL of the older adults in Tennessee and assess how oral health impacts their overall quality of life and their perception of dental well-being using the OHIP-14 questionnaire.

## 2. Methods

The quality of life surveys were distributed to Smile-on program patients through dental providers from December 2019 to August 2021. All participants aged 60 years or above and enrolled in the TN Smile-on program were included. There were 298 returned surveys. Only 233 surveys were valid for data analysis; surveys were excluded if they were not completed, the individuals were under 60 years old, or there were severe missing values. The patients completed a paper-based survey of dental providers. The dental provider shipped surveys to the investigator in a timely manner. The questionnaire contained items related to demographics and oral health-related quality of life. East Tennessee State University and Veteran Affairs (ETSU/VA) medical research Institution Review Board (IRB) approved this study with Approval Code: 0119.15e on 26 November 2019.

Oral Health Impact Profile included 14 questions (OHIP-14) with one-year reference. The five-point Likert scale was used with the following values: Very often = 4, fairly often = 3, occasionally = 2, and hardly ever = 1, never = 0. The sum was obtained by adding each response. The sum ranged from 0 to 56. Higher OHIP scores indicated poor self-reported oral health.

Demographic variables included in this study were age (60–70 years, >70 years), gender (men and women), race (Alaska Native, Asian, Black, Hispanic, and white), marital status (married or living with a partner (reference), never married, divorced/separated, widowed), degree (<high school diploma, high school diploma, college education), employment status (working, retired, unable to work)

The frequency and percentage for each sub-group were calculated. The central tendency (median) and variability (interquartile IQR) were calculated. The normal distribution of OHIP_sum was investigated with four different normality tests, which included the Shapiro–Wilk test, Kolmogorov–Smirnov test, Cramer–von Mises and Anderson–Darling test, consistently indicated both OHIP_sum scores are not normally distributed. Non-parametric Mann–Whitney’s U was used to test two groups difference, and the Kruskal–Wallis test was used to detect the overall significance for three or more groups.

Cronbach’s alpha was used to measure the internal consistency or reliability of OHIP in this study. Higher correlations, that is, larger Cronbach’s alpha, suggest that the item is more consistent with the overall scale. An alpha greater than 0.7 or higher is usually considered acceptable [25,26]. To address instrument reliability, item-to-total correlations for each question were calculated, and the corrected item-to-total correlation > 0.4 indicates very good discrimination [27].

In this study, factor analysis (FA) with oblique rotation was conducted to explore the underlying factor structure of the OHIP questionnaire set, assuming the presence of correlations among these items [28]. An individual item’s factor loading was considered substantial if its absolute value exceeded 0.50. Kaiser’s measure was used to assess the measure of sampling adequacy (MSA), with values between 0.8 and 1 indicating adequacy [29]. All statistical analyses were performed using the Statistical Analysis System (SAS, version 9.4, Cary, NC, USA), with a significance level of 0.05.

## 3. Results

Table 1 provides a breakdown of the OHIP_sum scores across various demographic and socioeconomic variables among older adults in Tennessee. The age range was from 60 to 90 years and the mean was 67.92 years (±s.d. 6.26 years). The participants are predominantly aged between 60 and 70 years (64.81%), with a smaller proportion aged 70 years and above (35.19%). Gender distribution shows a higher percentage of women (63.09%) compared to men (36.91%), and the OHIP_sum scores for men and women are 17 and 15, respectively. Racial demographics indicate that the majority of participants are white (84.55%), followed by Black (12.02%), with other races, including Hispanic and Asians, making up a very small percentage (<5%) of the sample. Marital status reveals that more than half of the participants are either married or living with a partner (33.48%) or widowed (24.46%), while educational attainment shows that over half of the participants have some college education (50.21%). The majority of participants are retired (59.66%), and there is a significant difference in OHIP_sum scores among different employment statuses (*p* = 0.018), indicating a potential impact of employment status on the OHRQoL.

Table 2 provides the Cronbach’s alpha values if each item is deleted for both raw and standardized variables. Correlation with the total score indicates how well an individual item correlates with the total score. Higher correlations suggest that the item is more consistent with the overall scale. It is noticed that correlation with the total appeared very similar for both the raw variables and standardized variables. Specifically, the domains of psychological discomfort (questions 5 and 6), physical disability (questions 7 and 8), psychological disability (questions 9 and 10) are highly correlated with the total score, with correlation coefficient around 0.8. More specifically, Question 6, “Have you felt tense because of problems with your teeth, mouth or dentures?” has the highest correlation with the total score (0.8064 for raw variables, 0.8016 for standardized variables). Item 9 “Have you found it difficult to relax because of problems with your teeth, mouth or dentures?” has the highest alpha if deleted value (0.944 for raw variables, 0.9448 for standardized variables), indicating that its removal would slightly improve the scale’s reliability. The domain of functional limitation (Questions 1 and 2) has a relatively small correlation coefficient: 0.54 for Question 1 and 0.66 for Question 2.

Alpha-if-Deleted shows what the overall Cronbach’s alpha would be if the specific item was removed from the scale. The values of Alpha-if-Deleted are consistent high around 0.95 in this study regardless using raw variable or standardized variable. Overall Cronbach’s alpha values are 0.95 for raw variables and 0.951 for standardized variables, indicating high reliability.

**Figure 1:**
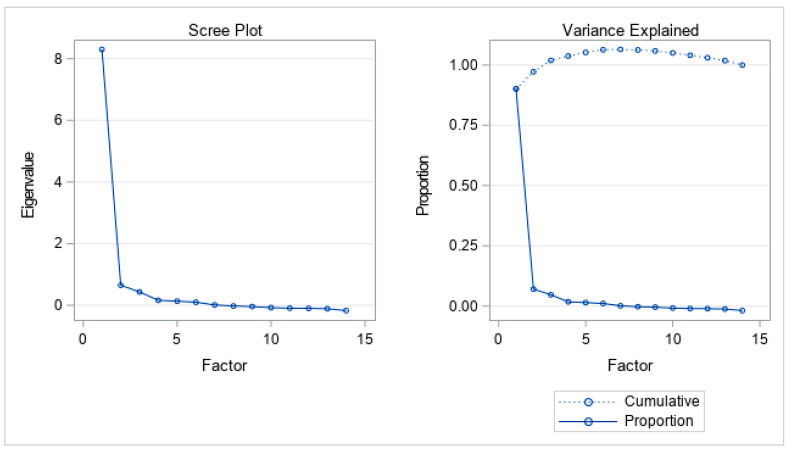
Three factors retained by the proportion criterion.

Table 3 lists variable loadings for each factor from a factor analysis with oblique rotation. Oblique rotation allows for the factors to be correlated, providing a more realistic representation of the data structure in cases where the underlying factors are not completely independent. Factor analysis found three factors with eigenvalues larger than 1. The loadings of each item on the three factors were identified by factor analysis (Figure 1). The three factors could explain over 90% of the total variance.

The detailed breakdown of each factor can be seen in Table 3. The result of factor analysis indicated that three underlying factors would represent the data. The evidence can be found in Figure 1, including a scree plot and the plot of variance explained. Individual MSA and overall MSA are greater than 0.9, which indicates excellent sampling adequacy (Table 4). Factor 1 is primarily associated with physical discomfort or dietary issues. Question 8, “Have you had to interrupt meals because of problems with your teeth, mouth or dentures?” has the highest loading (0.90), followed by Question 4, “Have you found it uncomfortable to eat any foods because of problems with your teeth, mouth or dentures?” (0.85) and Question 7, “Has your diet been unsatisfactory because of problems with your teeth, mouth or dentures?” (0.82). Factor 2 mainly encompasses psychological aspects and self-consciousness. Question 5, “Have you been self-conscious because of your teeth, mouth or dentures?” has high loading (0.87), followed by Question 10, “Have you been a bit embarrassed because of problems with your teeth, mouth or dentures?” (0.85). The loading of Question 6, “Have you felt tense because of problems with your teeth, mouth or dentures?” (0.62) is moderate. Factor 3 focuses on functional limitations and overall disability. The questions were found to have high loading on Factor 3. They are Question 12, “Have you had problems doing your usual jobs because of problems with your teeth, mouth or dentures?” (0.76), Question 14, “Have you been totally unable to function because of problems with your teeth, mouth or dentures?” (0.72), and Question 11, “Have you been a bit irritable with other people because of problems with your teeth, mouth or dentures?” (0.62).

## 4. Discussions

This study assessed the oral health outcomes of the population in Tennessee aged 65 years and above using OHIP-14, which measures seven domains of oral health-related quality of life, which include functional limitation, physical pain, psychological discomfort, physical disability, psychological disability, social disability, and handicap. It is interesting to note that, at an overall level, age, gender, race, marital status, and education were not found to be statistically significant. However, notable differences in magnitude were observed in this study—for instance, Black individuals exhibited higher OHIP_sum scores compared to white individuals, and divorced individuals had the highest OHIP_sum scores among all marital status groups. Additionally, individuals who were currently employed showed higher OHIP_sum scores than retirees. These findings suggest that future studies are warranted to further investigate these disparities. The OHIP-14 showed strong reliability in measuring oral health-related quality of life (OHRQoL). In terms of the difference in OHRQoL among various demographic groups, a previous study on factors influencing OHRQoL in older adults showed that OHRQoL differed according to educational level and monthly income, with lower education and lower income being associated with poorer OHRQoL [30]. Another study highlighted no significant differences in OHIP–14 scores between genders. However, it revealed significantly lower scores among individuals with academic education, dentate subjects, and those who use prosthetic appliances [31].

Although the OHIP-14 is a widely used and validated instrument for assessing oral health-related quality of life, we conducted factor analysis to evaluate its construct validity specifically within our study population, older adults in Tennessee enrolled in the Tennessee Senior Smiles program. Given potential cultural, regional, and demographic differences, factor structures can vary across populations. Conducting factor analysis allowed us to confirm whether the established dimensions of the OHIP-14 were consistent with the data from our sample or if any deviations existed. The factor analysis in this study identified the underlying structure of the data by examining how the OHIP-14 items correspond to different dimensions of OHRQoL. The results revealed that oral health issues among the study population can be categorized into three main domains: (1) physical discomfort and functional limitations, (2) psychological discomfort and social impacts, and (3) functional and social disability. According to the WHO model of health, the effects of oral disease are interconnected, with impairments, such as structural abnormalities (e.g., caries, periodontal diseases, or oral cancers), leading to functional limitations (e.g., difficulty chewing, speech difficulties, or restricted mouth opening) and pain/discomfort. These, in turn, can contribute to emotional distress and social embarrassment, ultimately affecting individuals’ ability to perform daily activities [32].

Although some studies have described oral health quality of life as a single construct in the older population, other reports have identified from three to five dimensions/domains related to physical, psychological, and social impacts [33]. Previous research performed in Germany by John et al. using the extended version of OHIP reported four dimensions: psychosocial impact, orofacial pain, oral functions, and appearance [34]. Similar to the findings in our study, Montero et al. found a three-factor structure (Psychosocial Impacts, Pain-Discomfort, and Functional Limitation) of the OHIP-14 through exploratory factor analysis [33].

The functional dimension of OHRQoL has been described to significantly affect an individual’s ability to eat and enjoy a wide range of dietary options. In addition to this, the functional limitation as a result of dental diseases has also contributed to sleep problems related to orofacial pain [35]. Both dental and systemic diseases can profoundly affect appetite and nutritional uptake, compromising overall health and well-being, especially in older people, where chronic illness and polymedication use are more common [35,36]. This study highlighted the social and psychological impacts of dental diseases on self-reported OHRQoL, which may be linked to conditions commonly found in the older population, such as extensive tooth loss as a result of periodontal diseases, temporomandibular disorders, and craniofacial deformities resulting from oral and pharyngeal cancers, among others. The psychological distress linked to oral health issues can contribute to avoiding social contact as a result of concerns over facial appearance, the isolating and depressing effects of chronic pain [37]. Additionally, social isolation or loneliness may occur as a result of the challenges associated with mastication, phonation, or communication or aesthetic concerns [38].

Older people represent a particularly vulnerable cohort of dental patients due to a combination of physical, cognitive, and psychosocial factors. Age-related health conditions, such as dementia, significantly impact their ability to maintain oral hygiene and communicate dental needs effectively. With the number of individuals affected by dementia projected to rise globally, this issue is set to become even more pressing [39]. Furthermore, older adults are at increased risk of abuse and neglect—both of which may go unnoticed, particularly in clinical settings where signs can be subtle or misattributed to aging. Psychosocial challenges, including isolation, depression, and limited access to care, further compound their vulnerability, underscoring the need for a sensitive and holistic approach in dental care for older adults [40].

The COVID-19 pandemic was marked by widespread disruptions to daily life, healthcare access, and psychological well-being. The pandemic likely significantly impacted participants’ quality of life, including their oral health-related quality of life. Lockdowns, reduced access to dental services, heightened stress, and changes in oral hygiene routines could have negatively influenced participants’ perceptions and experiences. In addition, the pandemic’s effect on stress and anxiety led to a decrease in health-promoting behaviors supporting oral disease prevention and patients postponing treatment out of fear of COVID-19 exposure. The restrictions on non-urgent care during the lockdown also influenced the use of dental services [41]. The closure of dental practices to non-urgent care led to a detrimental interruption to regular and preventive care leading to an increase or worsening of oral health conditions such as cavities and periodontal diseases [42]. This context represents a strong confounding factor, as it may obscure or exaggerate the relationships between the studied variables. For example, declines in oral health-related quality of life might not solely reflect the effects of the variables under investigation, but also pandemic-related stressors and limitations. Future studies should consider the potential influence of such external events when interpreting results, and, where possible, adjust for these factors statistically or through study design.

The convenience sampling method used in this study can be considered a limitation. In addition, the use of a general sample of dental patients, without specifying a clinical condition, can also be considered a limitation since, as previously mentioned, the clinical condition can affect how the instrument functions, and it can also provide information on how different oral diseases or conditions affect an individual’s quality of life. This study’s sample was limited to a specific age group (≥60 years) and so cannot be generalized. Data were collected during the COVID-19 pandemic, which limited the potential participation, and limited the number of surveys collected.

## 5. Conclusions

Despite limitations related to sampling and data collection during the COVID-19 pandemic, the findings of this study provide valuable insight into the oral health needs of older Tennesseans and offer a foundation for future research and program development aimed at enhancing OHRQoL in older adults.

## Figures and Tables

**Table 1:** The characteristics of the study population and OHIP_sum scores by demographic variable.

N = 233			**OHIP_Sum Scores**	
	**Frequency**	**Percent**	**Median (IQR)**	***p***
**Age**				0.125
60 ≤ age < 70 (reference)	151	64.81	17(26)	
≥70	82	35.19	15(21)	
**Gender**				0.895
Men (reference)	86	36.91	17(23)	
Women	147	63.09	15(26)	
**Race**				0.123
White (reference)	197	84.55	16(23)	
Alaska Native	1	0.43		
Asian	4	1.72	3(0)	
Black	28	12.02	26(24)	
Hispanic	1	0.43		
Refused	2	0.86	5.5(5)	
**Hispanic**				0.79
No (reference)	223	95.71	17(24)	
Yes	7	3	12(26)	
Refused	3	1.29	8(27)	
**Marital status**				0.287
Married or living with a partner (reference)	78	33.48	15.5(25)	
Never married	26	11.16	8.5(25)	
Divorced/separated	71	30.47	21(25)	
Widowed	57	24.46	17(26)	
Refused to answer	1	0.43	N/A	
**Degree**				0.091
<high school diploma (reference)	32	13.73	17(23.5)	
High school diploma	76	32.62	12(24.5)	
College education	117	50.21	18(26)	
Refuse to answer	8	3.43	7(18.5)	
**Employment**				**0.018**
Working (reference)	37	15.88	19(27)	
Retired	139	59.66	13(22)	
Unable to work	37	15.88	26(19)	
Refused	20	8.58	12.5(26)	

Note: IQR is interquartile range. *p*-values were from non-parametric Mann–Whitney’s U test for two groups difference, and Kruskal–Walis test for three or more groups.

**Table 2:** Cronbach coefficient alpha with deleted variable.

		Raw Variables	Standardized Variables		
	Deleted Variable	Correlation with Total	Aplha	Correlation with Total	Aplha
**Functional** **Limitation**	1. Have you had trouble pronouncing any words because of problems with your teeth, mouth or dentures?	0.5425	0.9515	0.5455	0.9523
	2. Have you felt that your sense of taste has worsened because of problems with your teeth, mouth or dentures?	0.6595	0.9486	0.6633	0.9494
**Physical ** **Pain**	3. Have you had painful aching in your mouth?	0.6936	0.9478	0.6926	0.9487
	4. Have you found it uncomfortable to eat any foods because of problems with your teeth, mouth or dentures?	0.7926	0.9454	0.7827	0.9464
**Psychological** **Discomfort**	5. Have you been self-conscious because of your teeth, mouth or dentures?	0.7573	0.9464	0.7468	0.9473
	6. Have you felt tense because of problems with your teeth, mouth or dentures?	0.8064	0.9449	0.8016	0.9460
**Physical Disability**	7. Has your diet been unsatisfactory because of problems with your teeth, mouth or dentures?	0.8346	0.9441	0.8285	0.9453
	8. Have you had to interrupt meals because of problems with your teeth, mouth or dentures?	0.8115	0.9448	0.8101	0.9457
**Psychological Disability**	9. Have you found it difficult to relax because of problems with your teeth, mouth or dentures?	0.8490	0.9440	0.8482	0.9448
	10. Have you been a bit embarrassed because of problems with your teeth, mouth or dentures?	0.7893	0.9454	0.7802	0.9465
**Social** **Disability**	11. Have you been a bit irritable with other people because of problems with your teeth, mouth or dentures?	0.7115	0.9474	0.7215	0.9480
	12. Have you had problems doing your usual jobs because of problems with your teeth, mouth or dentures?	0.7020	0.9478	0.7147	0.9481
**Handicap**	13. Have you felt that life in general was less satisfying because of problems with your teeth, mouth or dentures?	0.7822	0.9456	0.7815	0.9465
	14. Have you been totally unable to function because of problems with your teeth, mouth or dentures?	0.6674	0.9489	0.6781	0.9490
Overall			0.95		0.951

**Table 3:** Variable loading for each factor from factor analysis with oblique rotation.

	Factor 1	Factor 2	Factor 3
**8**. Have you had to interrupt meals because of problems with your teeth, mouth or dentures?	**0.90**	−0.11	0.10
**4**. Have you found it uncomfortable to eat any foods because of problems with your teeth, mouth or dentures?	**0.85**	0.19	−0.19
**7**. Has your diet been unsatisfactory because of problems with your teeth, mouth or dentures?	**0.82**	0.11	−0.03
**3**. Have you had painful aching in your mouth?	0.52	0.12	0.14
**9**. Have you found it difficult to relax because of problems with your teeth, mouth or dentures?	0.41	0.28	0.28
**1**. Have you had trouble pronouncing any words because of problems with your teeth, mouth or dentures?	0.41	−0.02	0.23
**5**. Have you been self-conscious because of your teeth, mouth or dentures?	0.04	**0.87**	−0.05
**10**. Have you been a bit embarrassed because of problems with your teeth, mouth or dentures?	0.02	**0.85**	0.04
**6**. Have you felt tense because of problems with your teeth, mouth or dentures?	0.13	**0.62**	0.18
**13**. Have you felt that life in general was less satisfying because of problems with your teeth, mouth or dentures?	0.11	0.50	0.31
**12**. Have you had problems doing your usual jobs because of problems with your teeth, mouth or dentures?	0.08	0.04	**0.76**
**14**. Have you been totally unable to function because of problems with your teeth, mouth or dentures?	0.08	0.02	**0.72**
**11**. Have you been a bit irritable with other people because of problems with your teeth, mouth or dentures?	−0.02	0.27	**0.62**
**2**. Have you felt that your sense of taste has worsened because of problems with your teeth, mouth or dentures?	0.38	0.05	0.32

**Table 4:** Kaiser’s measure of sampling adequacy.

1. Have you had trouble pronouncing any words because of problems with your teeth, mouth or dentures?	0.96378
2. Have you felt that your sense of taste has worsened because of problems with your teeth, mouth or dentures?	0.97215
3. Have you had painful aching in your mouth?	0.9398
4. Have you found it uncomfortable to eat any foods because of problems with your teeth, mouth or dentures?	0.9183
5. Have you been self-conscious because of your teeth, mouth or dentures?	0.90619
6. Have you felt tense because of problems with your teeth, mouth or dentures?	0.92722
7. Has your diet been unsatisfactory because of problems with your teeth, mouth or dentures?	0.94655
8. Have you had to interrupt meals because of problems with your teeth, mouth or dentures?	0.93094
9. Have you found it difficult to relax because of problems with your teeth, mouth or dentures?	0.96087
10. Have you been a bit embarrassed because of problems with your teeth, mouth or dentures?	0.9282
11. Have you been a bit irritable with other people because of problems with your teeth, mouth or dentures?	0.96009
12. Have you had problems doing your usual jobs because of problems with your teeth, mouth or dentures?	0.91001
13. Have you felt that life in general was less satisfying because of problems with your teeth, mouth or dentures?	0.92387
14. Have you been totally unable to function because of problems with your teeth, mouth or dentures?	0.94225
Overall MSA	0.93

## Data Availability

The data presented in this study are available upon reasonable request from the corresponding author.
